# A novel method to determine respiratory system mechanics during assisted ventilation

**DOI:** 10.1186/2197-425X-3-S1-A669

**Published:** 2015-10-01

**Authors:** D Schädler, T Becher, G Zick, I Frerichs, N Weiler

**Affiliations:** University Medical Center Schleswig-Holstein, Campus Kiel, Anaesthesiology and Intensive Care Medicine, Kiel, Germany

## Introduction

Knowledge of respiratory system mechanics during assisted ventilation is of high clinical interest. To date, it is only possible to measure it using an esophageal catheter or with the help of maneuvers using a specifically modified ventilator [1, 2].

## Objectives

To assess the accuracy of a novel method to determine respiratory system mechanics during pressure-support ventilation in a prospective clinical study.

## Methods

We included 20 mechanically ventilated patients. Respiratory system compliance was calculated by dividing the volume change induced by small changes in pressure-support level by the corresponding change in mean inspiratory airway pressure with subsequent adjustment for the pressure-support termination criterion (25% of peak inspiratory flow). To determine the respiratory time constant (τ), every expiratory flow-volume curve was divided into ten slices of equal volume. τ was calculated as the mean value of the curve´s slopes in every slice of one breath only in the breaths with linear flow-volume relationship. Respiratory system resistance was derived by dividing compliance by τ. As a reference, we determined respiratory system mechanics measured during volume-controlled ventilation with constant inspiratory flow.

## Results

Correlation analyses showed a good correlation of compliance (r2 = 0.74; bias -5 ml/cm H2O), a moderate correlation for τ (r2 = 0.68; bias 202 ms) and a poor correlation for resistance (r2 = 0.05; bias 11.8 cm H2O/l/s) when compared with the reference method.

## Conclusions

We conclude that the proposed novel, non-invasive method for the assessment of respiratory system mechanics in assisted ventilated patients delivered proper values for compliance and respiratory time constant but not for resistance. Determination of reliable resistance values might require the use of additional methods.
